# Paper-based microfluidic chip for rapid detection of SARS-CoV-2 N protein

**DOI:** 10.1080/21655979.2021.2014385

**Published:** 2021-12-30

**Authors:** Mingdi Sun, Man Han, Shengnan Xu, Kai Yan, Gul Nigal, Tongyang Zhang, Bo Song

**Affiliations:** Medical Technology College, Qiqihar Medical University, Qiqihar, China

**Keywords:** Paper-based microfluidic chip, N protein, p-ELISA, antigen detection, chromogenic response

## Abstract

This research has developed a method for rapid detection of SARS-CoV-2 N protein on a paper-based microfluidic chip. The chitosan-glutaraldehyde cross-linking method is used to fix the coated antibody, and the sandwich enzyme-linked immunosorbent method is used to achieve the specific detection of the target antigen. The system studied the influence of coating antibody concentration and enzyme-labeled antibody concentration on target antigen detection. According to the average gray value measured under different N protein concentrations, the standard curve of the method was established and the sensitivity was tested, and its linear regression was obtained. The equation is y = 9.8286x+137.6, R2 = 0.9772 > 0.90, which shows a high degree of fit. When the concentration of coating antibody and enzyme-labeled antibody were 1 μg/mL and 2 μg/mL, P > 0.05, the difference was not statistically significant, so the lower concentration of 1 μg/mL was chosen as the coating antibody concentration. The results show that the minimum concentration of N protein that can be detected by this method is 8 μg/mL, and the minimum concentration of coating antibody and enzyme-labeled antibody is 1 μg/mL, which has the characteristics of high sensitivity and good repeatability.

## 1. Introduction

The new coronavirus causes an acute respiratory infectious disease in humans-severe acute respiratory syndrome (SARS), which poses a great threat to the health of patients around the world [1–3].The naming method of the International Committee on Classification of Viruses is SARS-CoV-2 [4–6]. The new type of coronavirus (severe acute respiratory syndrome coronavirus 2,SARS-CoV-2) belongs to the genus of coronavirus β, and its genetic characteristics are significantly different from SARS-CoV and MERS-CoV [7]. The structural proteins of SARS-CoV-2 include S protein (spike glycoprotein), E protein (envelope glycoprotein), M protein (membrane glycoprotein), and N protein (nucleocapsid protein). N protein is the core protein of SARS-CoV-2 in cell assembly which wraps SARS- CoV-2 genomic RNA [8,9]. Its number is the largest and relatively conservative. Generally, human body produces anti-N protein antibody IgM in the early stage of SARS-CoV-2 infection, so N protein is one of the most important raw materials for SARS-CoV-2 serological detection [10,11]. The main route of transmission of the new coronavirus can be through respiratory droplets or through contact [12]. Because SARS-CoV-2 is generally susceptible to the population, early detection, early isolation, and early treatment can reduce the harm caused by SARS-CoV-2 to public health.

The most commonly used traditional methods for detecting viruses in clinical practice are nucleic acid detection and immunological detection [13]. Nucleic acid detection methods usually have high specificity and sensitivity, and are the gold standard for detection. However, it needs to be carried out in a zoned laboratory with conditions and qualifications, which requires high equipment and personnel operation and takes a long time. The immunological detection of antibody antigen method based on virus structural protein is fast and convenient.Compared with nucleic acid testing, it is easier to obtain samples and the testing time is shorter, which greatly reduces the risk of infection of medical staff during sampling and testing [14]. The incubation period of SARS-CoV-2 is generally 3 to 7 days. IgM antibodies are usually produced within a week, suggesting a recent infection. IgG antibodies appear later than IgM antibodies, but because IgG antibodies can exist in the body for a long time, the detection can distinguish acute infections from previous infections [15]. After the patient develops certain antibodies, immunological detection methods can be used. For example, IgM and IgG antibody detection kits based on enzyme linked immunosorbent assay (ELISA) [16] and colloidal gold detection method [17] are now on the market [18].

For SARS-CoV-2, immunological detection methods developed to detect virus-specific antibody antigens are mainly aimed at the structural proteins S protein and N protein with high immunogenicity. Studies have shown that recombinant S protein is more conducive to serological detection than recombinant N protein [19], but S protein is a highly glycosylated protein [20], and the success rate of expression of S protein in mammalian cells is limited, so N protein is commonly used for immunology diagnosis. The detection antigen of the new coronavirus antibody detection kits that have been approved for marketing are mainly N protein. The SARS-CoV-2 N protein can be detected by sandwich ELISA and quantum dot-based rapid immunochromatographic detection. Studies have found that the detection limit of ELISA and rapid immunochromatography both reach the ng level, which has high sensitivity [21]. The General Hospital of the Central Theater of the People’s Liberation Army also established a fluorescence immunochromatographic method to detect SARS-CoV-2 N protein in patients’ nasopharyngeal swabs and urine within 10 minutes, and found that this method has high sensitivity and specificity. It also can detect early patients after 3 days of fever [22,23]. This shows that N antigen determination is an accurate, rapid, early and simple method for diagnosing COVID-19, and it can be applied to large-scale screening [22].

The paper-based microfluidic chip (Microfluidic paper-based analytical device, μPADs) uses filter paper, chromatography paper, nitrocellulose membrane and other materials instead of traditional polymers, glass and other materials to treat the surface of the paper to form a hydrophilic/hydrophobic channel and A device for experiments in the zone is a new member of the microfluidic analysis system. Because of its wide range of sources, low cost, easy processing, good biological sample compatibility, and degradability, its carrier paper is often used as a base material for load-bearing analysis and diagnostic tests. Compared with traditional microfluidic chips, μPADs has low cost, simple preparation, no complicated peripheral equipment, and can perform truly one-time, low-cost, and portable analysis. Since the Whitesides team put forward μPADs in 2007, paper-based microfluidic technology has gradually become a research hotspot, and involves many fields such as medical diagnosis, environmental monitoring, and biochemical analysis. At present, paper-based microfluidic chips have become a new type of platform technology, which has shown great application potential in the field of rapid diagnostic testing in areas with low resource allocation [24].

The p-ELISA method (paper-based enzyme-linked immunosorbent assay, p-ELISA) used in this article combines the advantages of traditional ELISA and paper. It has been extensively studied because of its specificity, simplicity, rapidity, portability, and low cost. People’s concern, especially in the analytical fields of medical testing, environmental testing and food safety. First, this paper established a paper-based enzyme-linked immunosorbent assay on the chip and optimized its reaction conditions. Secondly, the paper-based microfluidic platform was used to realize the visual detection of N protein.Finally, the sensitivity of detecting SARS-CoV-2 N protein based on p-ELISA method was confirmed.

## 2. Establishment of SARS-CoV-2 paper-based enzyme-linked immunosorbent assay

### 2.1 Experimental Materials

#### 2.1.1 Main experimental reagents

Whatman 3 MM filter paper (200 mm×200 mm, purchased from Sigma), bovine serum albumin (BSA, A104912), New Crown N protein (gift from Guangzhou Qianxun Biotechnology Co., Ltd.), New Crown monoclonal antibody (purchased from Guangzhou Qianxun Biotechnology Co., Ltd.), Anti-mouse secondary antibody (purchased from Guangzhou Qianxun Biotechnology Co., Ltd.), diluent (0.01 mol/L phosphate buffered saline, PBS), washing solution (PBS containing 0.05% Tween-20, PBST), blocking solution (PBST solution containing 5% skimmed milk), color developing solution (TMB buffer) and other solutions: laboratory configuration.

#### 2.1.2 Main experimental instruments

Cutting machine (No.CE5000-40-CRP, Beijing Xingyun Shengshi Technology Co., Ltd.), washing machine, smart phone-used to take pictures, drawing software (Auto CAD 2013) used to design microfluidic channels, pictures are carried out with ImageJ software analyze.

### 2.2 Experiment content

#### 2.2.1 Design and preparation of paper-based microfluidic chips

**Figure 1.**
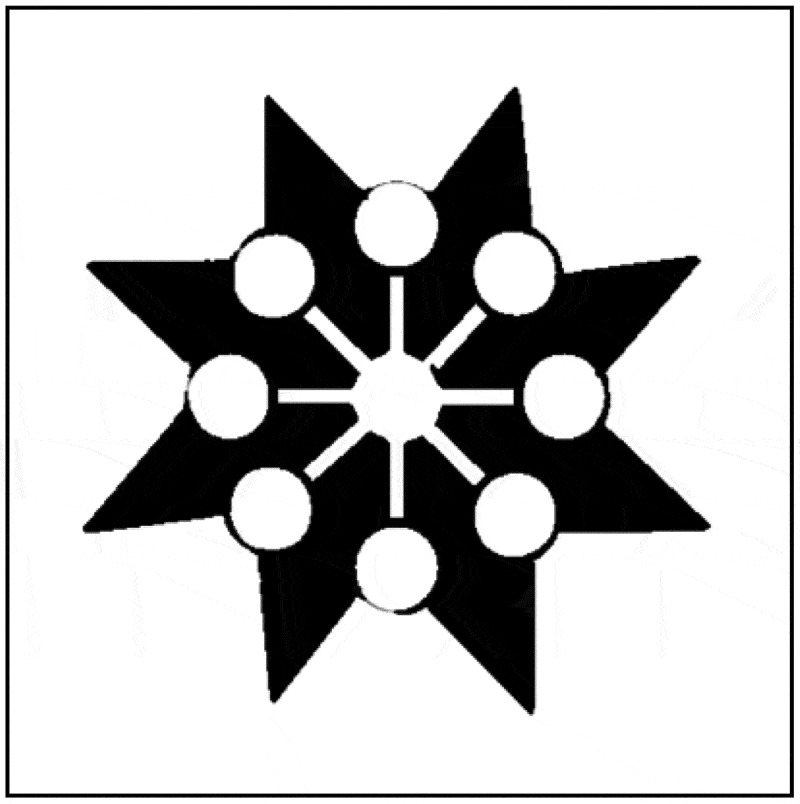
Mode of paper chip.

Designed with CoreldrawX6 vector drawing software, the chip is composed of eight detection areas, a sample injection area and a waste liquid area (Figure 1). Use the cutting software to open the pattern to be cut. Place Whatman 3 MM filter paper on a plastic plate, which is the carrier, and cover it with plastic film for cutting. Adjust the height and strength of the cutter head of the cutting machine to ensure that the paper chip can be cut off during the cutting process without damaging the plastic plate as the carrier. Adjust the starting position of the cutting appropriately so that the cutting knife starts cutting from the edge of the paper. Use a cutting machine (No.CE5000-40-CRP) to cut out a paper chip with a diameter of 50 mm. The chip is composed of 8 reaction areas with a diameter of 5 mm, a sampling area with a diameter of 5 mm, and a surrounding waste liquid area. The cut paper chips are placed in a clean box for later use [25].

#### 2.2.2 The establishment of double antibody sandwich paper-based enzyme-linked immunosorbent assay (p-ELISA)

**Figure 2.**
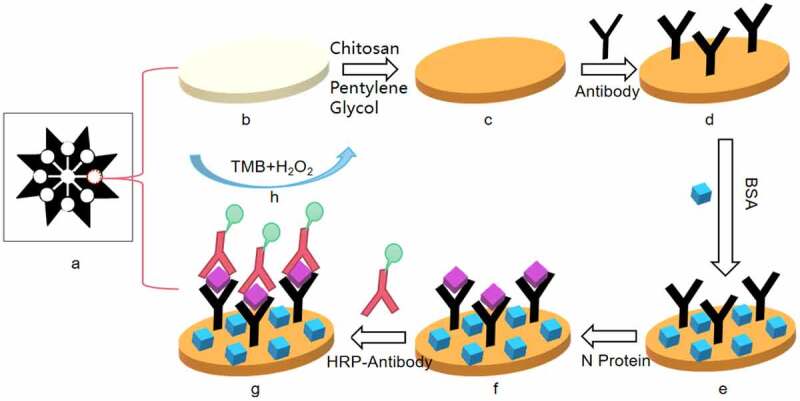
Schematic diagram of paper chip immunoassay experiment (a) paper chip made by cutting technology; (b) paper chip after dropping chitosan; (c) paper chip after dropping glutaraldehyde; (d) SARS-Cov-2 antibody fixed on the paper chip; (e) Add BSA to block the unbound antibody site of the paper chip; (f) Drop SARS-Cov-2 N protein into the detection area of the paper chip; (g) Drop HRP-labeled antibody; (h) Add TMB for color reaction.

The specific experimental steps of the sandwich enzyme-linked immunosorbent assay based on the microfluidic paper chip are shown in (Figure 2). Fix the antibody in the detection area of the microfluidic paper chip by chitosan-glutaraldehyde cross-linking method. The specific process is as follows: 2.5 μL of 0.25 mg/mL chitosan solution is added to each detection area. After reacting for 30 minutes at room temperature, 2.5 μL of 2.5% glutaraldehyde solution was added dropwise to each detection area. Then, after two hours of reaction, it was washed to remove the glutaraldehyde cross-linked on the paper surface. 2.5 μL of 20 μg/mL antibody was added dropwise to detect the area, and allow it to react for 30 minutes at room temperature, wash and remove the antibodies that are not fixed on the paper chip. Drop 2.5 μL of 2.5% BSA solution to the detection area to seal the unbound antibody sites on the paper chip to reduce nonspecific adsorption, react for 15 minutes, and wash to remove the unbound BSA on the paper chip. Add 2.5 μL of SARS-CoV-2 N protein standard solutions of different concentrations to different detection areas, and after fully reacting for 10 minutes, wash and remove the SARS-CoV-2 N protein that is reacted. Add 2.5 μL of HRP-labeled antibody to each detection area in turn and allow it to fully react for 15 minutes to form an antibody-antigen-enzyme-labeled antibody complex, and wash and remove the antibody that has not interacted with the SARS-CoV-2 N protein.Finally, add 2.5 μL of the substrate developed with HRP in Western blotting TMB. After the reaction is complete, use the camera to capture the color change of the paper chip after the reaction, and analyze the color intensity by Image J software. According to the color intensity and the SARS-CoV-2 N protein Concentration relationship for quantitative analysis. During each reagent dropping process, the paper chip is placed on a petri dish with a diameter of 30 mm to ensure that the sample area of the paper chip is suspended, which not only ensures the cleanliness of the paper chip, but also ensures that the sample will not spread randomly. After adding reagents, cover the paper chip to prevent the influence of suspended matter in the air on the experiment.

## 3. Results

In order to save reagents without affecting the color development of the experimental image, we optimized the concentration of the coated antibody and the concentration of the enzyme-labeled antibody. Then, the sensitivity and linear regression relationship of the established SARS-CoV-2 N double-antibody sandwich method were measured.

**Figure 3.**
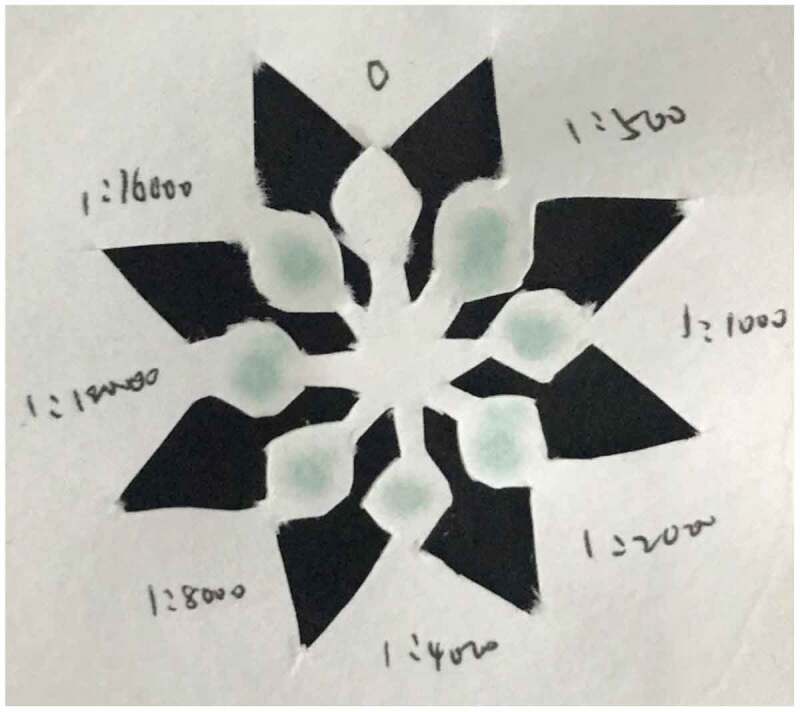
Color development results of SARS-Cov-2 N protein concentration of 133 μg/ml.

1. Directly observe the results with naked eyes: the darker the color in the reaction hole, the stronger the positive degree, and the negative reaction is colorless or extremely light. According to the depth of the color, it is indicated by ‘+’ and ‘-’ signs. Or use a smart phone to shoot the color-developing paper chip into JPEG format, import the photo into the computer, and use the Image J software to analyze the color intensity. Finally, make a ‘yes’ or ‘no’ answer to the result of the qualitative determination, which is represented by ‘positive’ and ‘negative’ respectively(Figure 3).

### 3.1 Optimization results of coating antibody and enzyme-labeled antibody concentration

Dilute the SARS-CoV-2 N primary antibody and the enzyme-labeled antibody at a concentration of 1 mg/mL in a gradient to obtain the primary antibody and enzyme-labeled antibody concentrations of 2 μg/mL, 1 μg/mL, 0.5 μg/mL, 0.25 μg/ mL, 0.125 μg/mL, 0.0625 μg/mL, and add them to the p-ELISA plate. Perform follow-up operations according to the detection process. Note: The diluted enzyme-labeled antibody is cultured in a 37°C incubator for 1 hour [26]

**Figure 4.**
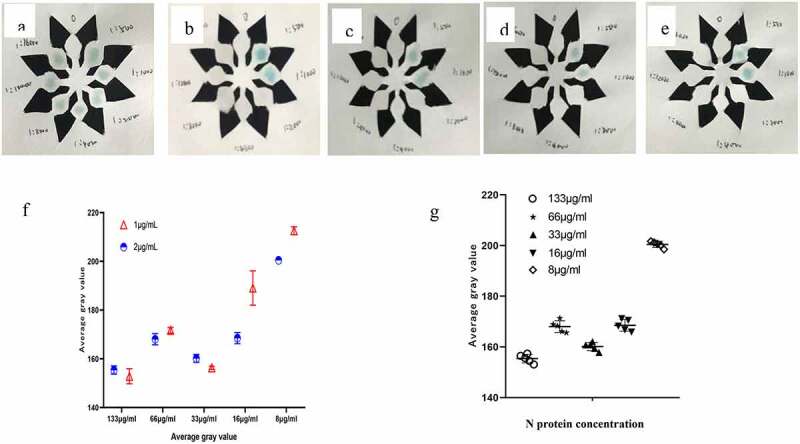
(a–e) The color development result when the N protein concentration is 133 μg/ml, 66 μg/ml, 33 μg/ml, 16 μg/ml, 8 μg/ml. f Comparison of average gray value at different N protein concentration(t = 1.069, P = 0.3163).g When the concentration is 1 μg/ml, the average gray value of N protein at different concentrations(F = 441.4, P < 0.001).

According to the experimental phenomenon (Figure 4), it can be concluded that when the concentration of coating antibody and enzyme-labeled antibody is 1 μg/mL, the results are good. Therefore, the concentration of coating antibody and enzyme-labeled antibody can be used at 1 μg/mL.

### 3.2 Establish the Standard Curve of SARS-CoV-2 N Double-antibody Sandwich Method

**Figure 5.**
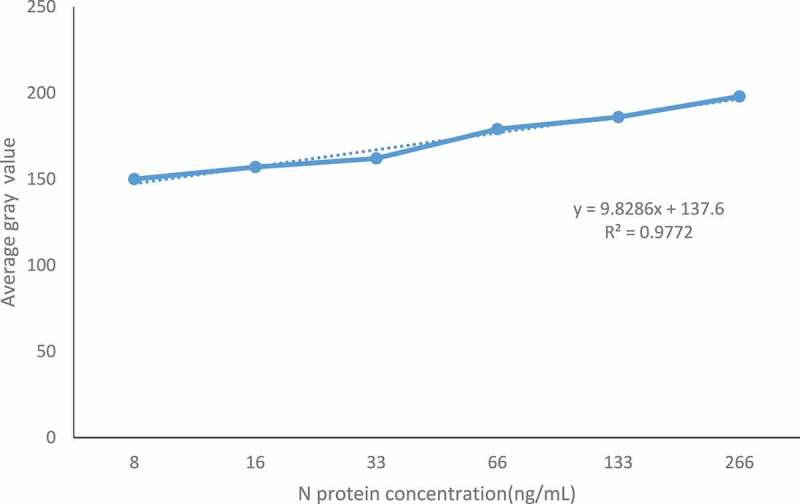
Standard curve of SARS-CoV-2 N double-antibody sandwich method.

Use diluent to dilute SARS-CoV-2 N antigen and set the following gradients: 8 μg/ml,16 μg/ml,33 μg/ml,66 μg/ml,133 μg/ml, 266 μg/ml, using the best experimental conditions found above complete the SARS-CoV-2 N p-ELISA detection process, record the experimental results and calculate the linear equation, and get the correlation coefficient. Establish a standard curve, and draw a linear regression equation in EXCEL according to the results, as shown in (Figure 5). When the concentration of SARS-CoV-2 N is between 0.008–0.266 mg/mL, the linear regression equation of SARS-CoV-2 N is y = 9.8286x+137.6, R^2^ = 0.9772.

### 3.3 Sensitivity determination

**Figure 6.**
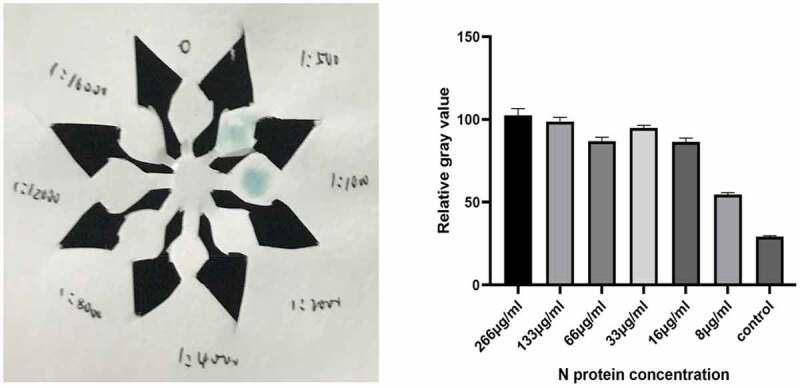
Color development results of SARS-Cov-2 N protein concentration of 8 μg/ml.

The SARS-CoV-2 N protein standard product (0.266 mg/ml) was diluted in steps to obtain N protein concentrations of 266 μg/mL, 133 μg/mL, 66 μg/mL, 33 μg/mL, 16 μg/mL, 8 μg/mL, to determine the sensitivity of the reagent. The SARS-CoV-2 N antigen was diluted in series and the SARS-CoV-2 N protein was determined by the p-ELISA detection method constructed above. The negative control was PBS, and SARS-CoV-2 was measured according to the cutoff value obtained in (1). The lowest detectable concentration of N antigen. As shown in (Figure 6), the minimum detectable concentration of SARS-CoV-2 N protein is 8 μg/mL.

## 4. Statistical analysis

All data were tested repeatedly for three times and expressed in the form of mean ± standard deviation. Statistical analysis was performed using SPSS19.0 statistical software. The comparison between the two groups was performed by t-test. P < 0.05 is considered to be between the different concentration of antigen coating and the intensity of color development. There is a significant correlation.

## 5. Discuss

The detection method in this paper is based on paper as a carrier, connected to a paper base with antibodies, antigens, and HRP-labeled enzyme-labeled antibodies to form an immune complex with a complex structure. For the luminescent substrate catalyzed by HRP, the gray value is measured after the catalysis is completed, and the quantitative analysis is carried out according to the relationship between the color intensity and the concentration of SARS-CoV-2 N protein. This detection method has high sensitivity and can shorten the detection time. In this paper, Image J software is used to analyze the color intensity to optimize each link in the experiment. Finally, 1 μg/ml antibody is used for coating, 2.5% BSA solution is used for blocking, and the concentration of enzyme-labeled antibody is 1 μg/mL. After adding the substrate, use the camera to capture the color change of the paper chip after the reaction. Use Image J software to analyze the color intensity to determine the gray value, and draw the standard curve. The linear regression equation is y = 9.8286x+ 137.6, R^2^ = 0.9772, when the SARS-CoV-2 N protein concentration is between 0.008–0.266 mg/mL, there is a linear relationship. Methodological evaluation of the constructed method showed that the established method has strong specificity, good reproducibility, and high sensitivity. It can detect SARS-CoV-2 N antigen at a minimum of 8 μg/ml without the ‘HOOK’ effect. Prove that the established method can be used for clinical testing.

The ELISA method is a commonly used detection method in laboratories and clinics. It has high sensitivity, good specificity, and is safe and pollution-free. The double antibody sandwich p-ELISA method we use combines the advantages of traditional ELISA and paper, and realizes the detection of SARS-CoV-2 N protein according to the change of color intensity after color development. Compared with the traditional enzyme-linked immunoassay, the whole experimental process requires less reagents, better washing method, low cost, simple steps, and it is a fast and simple detection method. According to reports, about 40% of the antibodies will dissociate during the washing process, which affects the sensitivity of detection. Most researchers improve sensitivity by improving antibody immobilization, enhancing detection signal, and reducing background signal. Although the above-mentioned problems still exist, it is foreseeable that the paper chip device proposed in this article has extremely high feasibility in the diagnosis of SARS-CoV-2 N protein, and can realize convenient and quick qualitative analysis, which provides a basis for the

application of paper chip in clinical detection of SARS-CoV-2 N protein. It has

certain clinical value. The detection of sensitivity of SARS-CoV-2 N protein were studied. This method can shorten the detection time of SARS-CoV-2 N protein. At the same time, the chip used in this experiment is small and easy to carry, while the traditional detection method requires large experimental equipment. It lays a portable preliminary experimental foundation for the further development of high sensitivity and rapid detection system.

## 6. Conclusion

In this paper, the detection of SARS-CoV-2 N protein using paper carrier is short in time and low in cost. The results showed that the microarray had a minimum concentration of 8 μg/mL for SARS-CoV-2 N protein, and was more sensitive and specific for SARS-CoV-2 N protein. The chip used in this experiment is small in size and more portable than the traditional microfluidic chip.

## Data Availability

All data generated or analyzed during this study are included in this published article.
